# The price of hope: CAR-T therapy in pediatric leukemia

**DOI:** 10.18632/oncoscience.598

**Published:** 2024-04-25

**Authors:** Alex Hoover

**Keywords:** CAR-T, cost, pediatric leukemia

We stand at the crossroads of medical innovation, where cutting-edge scientific discoveries intersect with the resilience of the human body, providing hope to families grappling with a diagnosis of pediatric leukemia. In the last decade, the chimeric antigen receptor T-cell (CAR-T) therapy tisagenlecleucel (tisa-cel) has been a groundbreaking development in the treatment of B-cell lineage acute lymphoblastic leukemia (B-ALL) – the most common childhood cancer. Following the pivotal ELIANA trial, tisa-cel was approved in the United States for the treatment of refractory or second or greater relapse of B-ALL in patients under age 25 [1, 2]. This innovative therapy involves genetically modifying a patient’s native T-cells – immune cells with the ability to kill and/or regulate infected or diseased cells – to express a receptor that targets cancer cells. The tisa-cel product is specifically engineered to target CD19 on B-lineage cells and has shown promising results in a historically challenging relapsed and refractory population.

Upon its approval in 2017, Novartis priced tisa-cel at $475,000 – a number that many healthcare systems and payers balked at initially – and in 2023 the price increased to $508,250. However, compared to more recently approved *ex vivo* gene therapies such as bluebird bio’s Zynteglo for beta thalassemia priced at $2.8 million or Vertex Pharmaceuticals’ Casgevy for sickle cell disease at $2.2 million, the half-million dollar tisa-cel price tag is less staggering. Importantly, the listed cost of these therapies includes only the product and does not include the healthcare cost for other necessary care including leukapheresis, chemotherapy administration and adverse event monitoring and management.

Our team recently published an in-depth analysis of the comprehensive cost and utilization of commercial CAR-T therapy in pediatric B-ALL patients with commercial insurance in the United States [3]. The analysis specifically focuses on a cohort of 37 patients, aged 1–25, who underwent CAR-T cell infusion between October 2016 and December 2021. Median total cost over the 90-day period surrounding product infusion was $620,500, with inpatient care accounting for about 71% of this cost. This study is significant as it provides the first real-world comprehensive cost report on CAR-T therapy for pediatric B-ALL. The data is crucial for further analyses regarding the cost-effectiveness of tisa-cel and offers valuable insights for healthcare systems managing the financial implications of such treatments.

Within the context of cost-effectiveness studies for patients with relapsed or refractory B-ALL, one essential consideration is that the only alternative treatment pathway to achieve long-term remission for most of these patients is allogeneic hematopoietic cell transplant (alloHCT). Analyses on the comprehensive cost of alloHCT in the modern era report a median total healthcare cost of $290,000–$330,000 [4, 5]. Although long term remission rates with tisa-cel alone are lower than those of HCT, there is significantly less toxicity compared to transplant and these factors are accounted for in robust cost-effectiveness studies. In addition, prior analyses based on algorithmically estimated cost reported that tisa-cel offered a competitive cost-effective approach [6–8]. With the real-world comprehensive cost we have now reported, future analyses may incorporate this data for more accurate modeling and conclusions.

Expanding on the implications of high-cost therapies particularly in the pediatric hematology/oncology context, treatments like CAR-T therapy bring forth fundamental questions about healthcare equity and access. The financial burden these therapies impose not only affects healthcare systems but also patients and their families, often leading to significant stress and financial hardship. Avoiding disparate access to potential life-saving measures based on socioeconomic status or financial advantage maintains pressure on the public safety net such as state Medicaid programs to find ways to cover these treatments. In pediatric oncology, where patients have their entire lives ahead of them, the ethical implications of health equity become even more profound. There is an inherent desire to provide the best possible care irrespective of cost, but this can be challenging for healthcare systems operating within financial constraints.

Furthermore, the evolution of cellular and gene therapies introduces a new paradigm in evaluating treatment effectiveness versus cost. While these therapies can be life-saving and potentially curative, their high upfront costs are a considerable hurdle. Commercial healthcare reimbursement systems in the United States are not designed to have particular foresight or perceive that the upfront cost for curative therapies may lead to lower longitudinal cost in the care of patients with otherwise chronic illnesses. The current payment structure for cellular and gene therapies is also complex and variable from state to state and center to center, further complicating the paradigm. This situation necessitates the exploration of new payment models, such as outcome-based payments or funding strategies that can alleviate the financial burden on patients and healthcare systems. The enormous cost will require financial restructuring and intensive collaboration between pharmaceutical companies, healthcare systems and payers.

The advancement of novel therapies like CAR-T in relapsed and refractory pediatric B-ALL is truly a beacon of hope for children and young adults with a previously dismal prognosis. However, it also brings to light critical challenges in drug pricing and healthcare financing and access, underscoring the need for innovative solutions to balance cost with the imperative to provide life-saving treatments to all who need them.
